# Mature Adrenal Ganglioneuroma With Lipomatous Content: A Radiological and Histopathological Diagnostic Challenge

**DOI:** 10.7759/cureus.75648

**Published:** 2024-12-13

**Authors:** Mohammed Lameir Hussein, Shams O Alkhateeb, Jouhar J Kolleri, Ala' Saleem Abu-Dayeh, Khaled Murshed, Nabil Sherif Mahmood

**Affiliations:** 1 Clinical Imaging Department, Hamad Medical Corporation, Doha, QAT; 2 Medical Education Department, Hamad Medical Corporation, Doha, QAT; 3 Pathology and Laboratory Medicine, Hamad Medical Corporation, Doha, QAT

**Keywords:** adrenal adenoma, adrenal gland incidentaloma, adrenal myelolipoma, ganglioneuroma, lipomatous changes

## Abstract

Adrenal incidentalomas are increasingly identified through advanced imaging, posing diagnostic challenges due to their varied benign and malignant nature. We present the case of a 29-year-old male who, during evaluation for left renal colic, was found to have a 5.5 cm heterogeneous right adrenal mass on non-contrast CT, initially suggestive of a myelolipoma. Subsequent contrast-enhanced CT supported this diagnosis. However, due to the size of the mass and potential complications, the patient underwent a robotic-assisted adrenalectomy. Histopathological examination unexpectedly revealed a 6.5 cm adrenal ganglioneuroma. This case highlights the difficulties in accurately diagnosing adrenal incidentalomas based solely on imaging, as the features of ganglioneuromas and myelolipomas can occasionally overlap significantly. Ultimately, while imaging plays a critical role in initial assessment, definitive diagnosis often requires histological analysis, underscoring the challenges posed by adrenal incidentalomas in clinical practice.

## Introduction

Adrenal incidentalomas are increasingly detected due to the widespread use of advanced imaging modalities like CT and MRI. These lesions are found in approximately 4-7% of imaging studies performed for unrelated reasons [1]. The differential diagnosis of adrenal incidentalomas includes a range of both benign and malignant conditions, including adrenal adenomas, myelolipomas, pheochromocytomas, ganglioneuromas, etc [2].

Adrenal myelolipomas are common benign lesions composed of fat and hematopoietic elements. Myelolipomas larger than 4 cm are at risk for complications such as hemorrhage or mass effect, warranting surgical resection in certain cases [3]. Adrenal ganglioneuromas, in contrast, are rare benign tumors derived from neural crest cells. Due to their slow growth and often asymptomatic nature, ganglioneuromas are typically found incidentally on imaging performed for unrelated complaints [4].

Here, we describe a case where an adrenal ganglioneuroma was initially misdiagnosed as a myelolipoma, emphasizing the challenges associated with an imaging-based diagnosis of adrenal incidentalomas.

## Case presentation

A 29-year-old male with no significant medical history presented with symptoms of left renal colic. A non-contrast CT scan of the abdomen, performed on May 2024, revealed a 2 mm left distal ureteric stone along with an incidental 5.5 cm right adrenal mass. The adrenal mass was heterogeneous, with areas of fat attenuation accounting for less than 50% of the tumor volume. An adrenal myelolipoma was suspected. Follow-up contrast-enhanced CT confirmed the mass measuring 4.6 x 6.5 x 7 cm with progressive enhancement, consistent with a benign adrenal lesion. The patient was normotensive and had no clinical signs of hormonal excess. Multidisciplinary team (MDT) discussion determined that robotic-assisted adrenalectomy was appropriate due to the size of the lesion and potential complications associated with mass effect. The surgery was performed on September 2024 without intraoperative complications.

**Figure 1:**
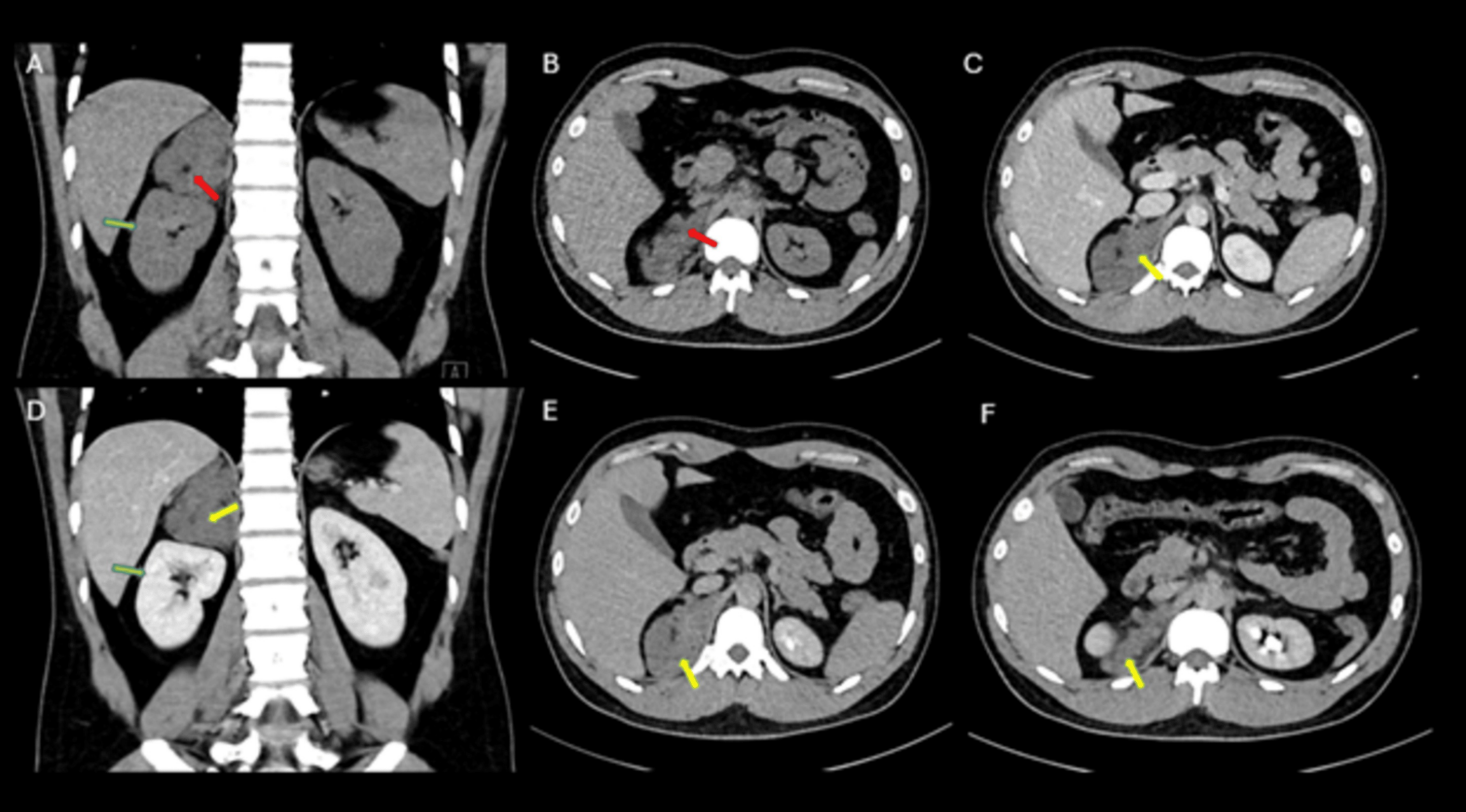
CT Abdomen and pelvis with IV contrast plain. A) coronal; B) axial, venous phase; C) axial; D) coronal, delayed phase; E) and F) showing right adrenal soft tissue mass with lobulated contour mostly arising from the medial limb (red arrows). There are intra-lesional macroscopic fat components with no calcification seen. On post-contrast images, there is progressive contrast enhancement (yellow arrows). The right kidney is displaced inferiorly by the adrenal mass with no invasion (green arrows).

A contrast-enhanced CT of the abdomen and pelvis revealed a lobulated right adrenal soft tissue mass, primarily originating from the medial limb, with intra-lesional macroscopic fat but no calcifications. Progressive enhancement was noted post-contrast. The mass displaced the right kidney inferiorly without evidence of invasion, while the left kidney and adrenal gland were unremarkable (Figure 1). These findings are suggestive of myelolipoma; however, another possibility of an adrenal neoplastic mass containing fat should be considered.

**Figure 2:**
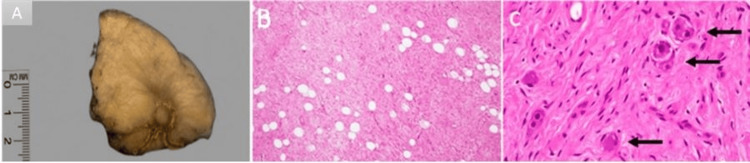
Excised mass and histopathological images. (A) gross photograph showing a well-demarcated mass with a firm gray-yellow cut surface. The residual adrenal gland is seen at the periphery of the mass; (B) the photomicrograph shows a tumor composed mostly of Schwannian stroma admixed with mature ganglion cells. Background includes mature adipose tissue (H&E stain x200); (C) high power view demonstrates scattered mature ganglion cells (black arrows) embedded within the wavy, fibrillary stroma  (H&E stain x400).

A robotic-assisted right adrenalectomy was performed. Intraoperatively, a well-circumscribed adrenal mass was noted. Histopathological analysis revealed a well-demarcated mass with a homogeneous firm gray-yellow cut surface measuring 6.5 cm (Figure 2A). The residual adrenal gland was seen at the periphery of the mass. Microscopic examination demonstrated a tumor composed mainly of Schwannian stroma admixed with mature ganglion cells. The background included mature adipose tissue (Figure 2B). The Schwannian stroma appeared fibrillary and eosinophilic, forming short fascicles with spindle cells showing elongated bland nuclei. The ganglion cells were mature, with granular eosinophilic cytoplasm, distinct cell borders, and a single eccentric round nucleus with a prominent nucleolus (Figure 2C). No neuroblastic component was identified. Surgical margins were negative for malignancy. The unexpected diagnosis of a ganglioneuroma rather than a myelolipoma highlighted the difficulty of differentiating between adrenal mass types based on imaging alone.

## Discussion

Ganglioneuromas are benign neurogenic tumors originating from the neural crest containing elements of both Schwannian and ganglion cell components. The condition is typically asymptomatic, non-invasive, and incidentally detected on imaging done for other reasons. Although ganglioneuromas can be found in the posterior mediastinum, retroperitoneum, and adrenal glands, among other various sites, adrenal ganglioneuromas represent a minority of these tumors [5].

Adrenal ganglioneuromas are often clinically silent, with patients rarely presenting with symptoms unless the tumor reaches a substantial size, causing mass effect or local compression. Despite their benign nature, the overlap of radiologic characteristics between ganglioneuromas and other adrenal incidentalomas, such as myelolipomas, adrenal adenomas, and pheochromocytomas, complicates preoperative diagnosis [2]. Imaging modalities such as CT and MRI are frequently used for evaluation, but they often cannot definitively distinguish between these lesions. For instance, adrenal ganglioneuromas may present as heterogeneous masses with variable degrees of calcification, fibrosis, and enhancement patterns on imaging, features that are also shared by other benign tumors like myelolipomas [6]. The proportion of fatty tissue in myelolipomas can vary widely. It may range from small, isolated areas within a predominantly soft tissue density mass (10%) to lesions with approximately equal amounts of fat and soft tissue (50%), or even to masses that are almost entirely composed of fat (40%) making it challenging to differentiate them from well-differentiated retroperitoneal malignancies or other adrenal tumors using CT or MRI scans [7].

Although extremely rare, ganglioneuromas and lipomatous alterations have been linked in the literature. The diagnostic confusion is exacerbated by lipomatous degeneration or the presence of adipose tissue within a ganglioneuroma, as these features can mimic imaging studies of liposarcomas or myelolipomas. Although myelolipomas exhibit a characteristic combination of fat and hematopoietic components, ganglioneuromas usually do not exhibit such substantial adipose components. Nonetheless, as some reports have indicated, the presence of adipose tissue within a ganglioneuroma may result from metaplastic changes, making this an uncommon histopathological finding [8].

Due to the presence of fat-containing areas on non-contrast CT, a characteristic common to myelolipomas, the patient's adrenal mass was initially diagnosed in the present case as a myelolipoma. Nevertheless, additional imaging and subsequent histological analysis showed the presence of a 6.5 cm ganglioneuroma. This case emphasizes how difficult it can be to distinguish between adrenal lesions just by imaging, particularly in the presence of unusual characteristics like lipomatous changes [9].

Adrenal ganglioneuromas are histologically composed of fibrous tissue, Schwann cells, and mature ganglion cells; there is no indication of malignancy or immature neuroblasts. When lipomatous elements are present, they are typically incidental and not the tumor's main characteristic. The pathogenesis of this association is still unknown, but these results imply that ganglioneuromas with lipomatous differentiation may represent a unique histopathological variant [10]. Some studies have hypothesized that lipomatous changes may result from metaplastic transformation of the stromal component or secondary degeneration, but the exact mechanism has not been fully elucidated [11].

Given the rarity of adrenal ganglioneuromas and their propensity for lipomatous differentiation, histological confirmation is essential in cases of large or indeterminate adrenal masses. Imaging tests are necessary for the preliminary assessment, but for a definitive diagnosis, surgical excision and histological examination remain the gold standard. Because of the potential for complications like bleeding or mass effect, which may necessitate resection even in cases where cancer is unlikely, this technique is especially important for larger tumors [12].

Adrenal ganglioneuroma surgery is usually curative, and with total resection, the prognosis is good, with a low chance of recurrence. In comparison to traditional open surgery, robotic-assisted adrenalectomy, as carried out in this instance, offers a less invasive technique with fewer postoperative problems and a quicker recovery [13]. The use of robotic techniques has been shown to be effective in the management of large adrenal tumors, providing precise dissection and reducing the risk of injury to surrounding structures [14].

## Conclusions

This case highlights the diagnostic complexities associated with adrenal incidentalomas, particularly when atypical features such as lipomatous changes are present. Ganglioneuromas, though benign, can mimic other adrenal lesions on imaging, necessitating surgical resection for definitive diagnosis. The rare occurrence of lipomatous differentiation in ganglioneuromas adds an additional layer of diagnostic difficulty, emphasizing the need for histological analysis to guide appropriate clinical management.
